# Supplementary figures and images for: Rising slopes—Bibliometrics of mountain research 1900–2019
Source: PLoS One. 2022 Aug 25;17(8):e0273421. doi: 10.1371/journal.pone.0273421 (PMC9409586; doi:10.1371/journal.pone.0273421)

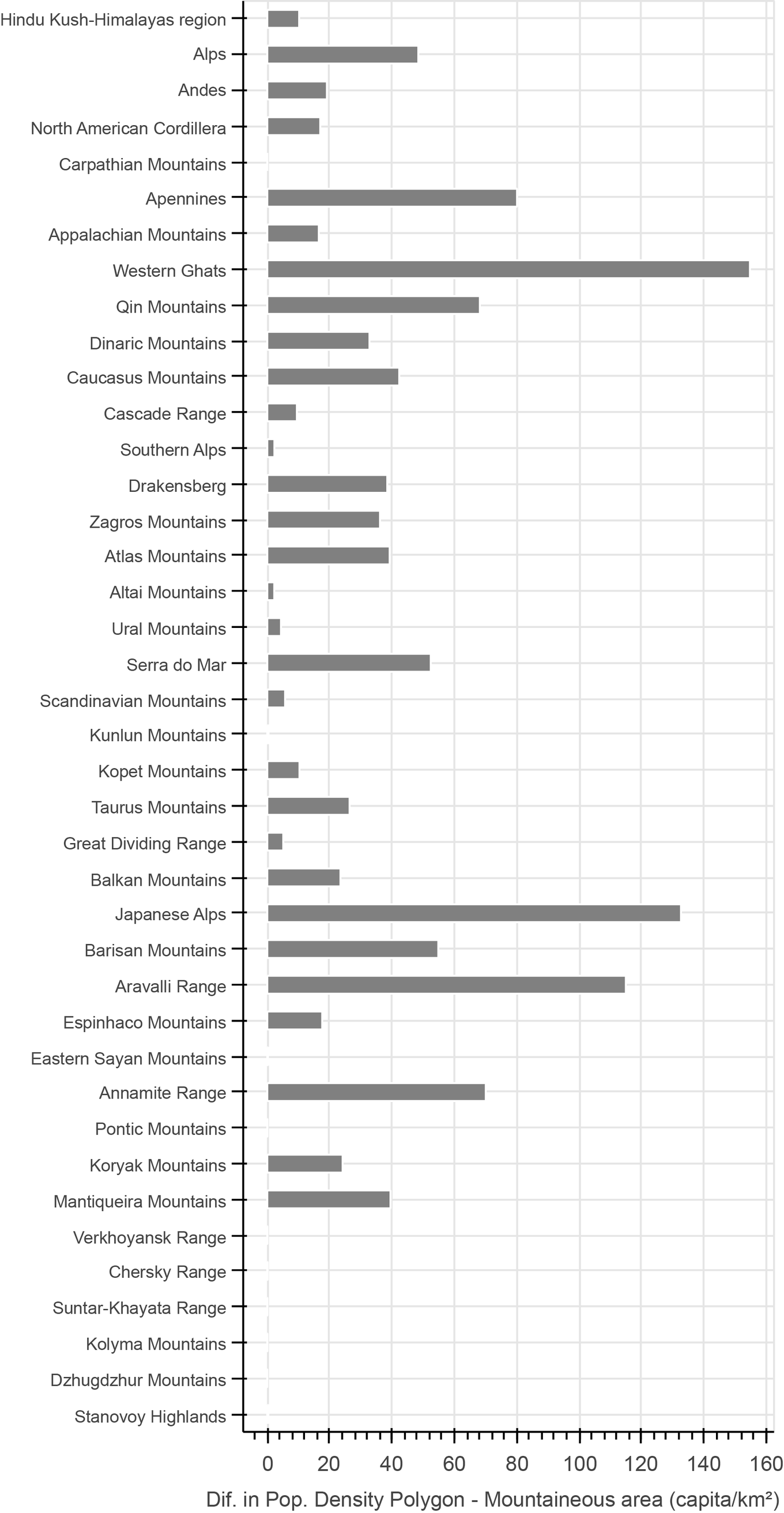

Supplement: S2 Fig — Difference in population density of mountain ranges calculated for the entire polygon area and only for the rugged terrain within the polygon area. (TIF) [file pone.0273421.s009.tif]
