# Supplementary material for: Rising slopes—Bibliometrics of mountain research 1900–2019
Source: PLoS One. 2022 Aug 25;17(8):e0273421. doi: 10.1371/journal.pone.0273421 (PMC9409586; doi:10.1371/journal.pone.0273421)
Supplement: Attachment — Submitted filename: ResponsestoReviewers_final.pdf [file pone.0273421.s011.pdf]

PONE-D-22-09392
Rising slopes - bibliometrics of mountain research 1900-2019

PLOS ONE

Dear editor,

we thank you and the reviewers for reviewing our manuscript. All comments seemed valuable to us and therefore, we have addressed all of them (in the manuscript) as described in detail below. The most important changes relate to the connection of our findings with very recent literature and the better description of the selected approach and potential benefits for upcoming investigations.

We tried to highlight most changes with the track change mode to facilitate the checks.

Thanks again for all your efforts!
The team of authors

01.06.2022

Reviewer #1:

Thank you for the opportunity to review the manuscript titled 'Rising slopes - bibliometrics of mountain research 1900-2019'.

I have one substantive recommendation for improvement, as well as a few minor points for the authors' consideration:

1. The main blind-spot in the manuscript is the lack of an introductory paragraph in the methods section that provides background on the chosen review method. For example, why was a bibliometric analysis selected over a systematic scoping review, how does such an approach differ, and why was it the most appropriate option given your study objectives? Adding such an introductory paragraph will round out an otherwise excellent methods section. This is my substantive recommendation.

We fully agree with the reviewer and have incorporated the motivation for doing a bibliometric analysis in the beginning of section 2 (lines 95-103).

2. The methods section does not mention a specific focus on 'peer-reviewed' articles/reviews. While this is indicated elsewhere in the manuscript, it seems prudent to state in the methods section.

Thanks for pointing this out, we added the information in line 93.

3. A map depicting mountain areas globally based on the criteria described in lines 222 – 230 would provide helpful geospatial context, particularly for readers not familiar with the global distribution of mountains. Depicting the 40 mountain ranges examined in your analysis would likely be too visually complex but could be considered. Perhaps such a map could be added at line 235.

We added an interactive map to the supplement (a respective hint is provided in lines 239-240). We prefer an interactive html version of the figure in the supplement over a static figure in the manuscript for perfect representation and visibility.

4. The phrase “economic power”, which appears throughout the manuscript, is slightly off putting. Could another word replace ‘power”, perhaps “capacity”?

We thank the reviewer for raising awareness and providing a good alternative. This was changed throughout the manuscript.

5. The observed paucity of research in the social sciences seems highly consequential, yet no specific call for more engagement with the social sciences in mountain research is provided in the Discussion and Conclusion section (lines 416-417). Perhaps it would be germane to include a line about the potential implications of this research gap, as well as the need to support such research in mountainous contexts?

We agree with the reviewer and have added respective information in the discussion.

6. Line 420-421 rightly suggest that it “is highly desirable that relevant findings are (also) published in English-language journals.” Should this recommendation also be accompanied by a call for the mountain research community/mountain organizations to provide support for such publishing (e.g. the establishment of a fund to support translation services)?

Good idea – we added this suggestion in the discussion.

Reviewer #2:

Suggest to address several minor comments, which have been uploaded as a separate attachment and are also listed below:

1. p.1, line 24: replace “would be desirable” with “is recommended”.

Changed accordingly.

2. p.2, lines 63-64: for comprehensive synthesis reports please consider also citing the HKH Assessment report (Wester et al., 2019) and the Cross-Chapter Paper Mountains in IPCC AR6 WGII (Adler et al., 2022).

These references have now been added.

3. p.9, lines 274- 276: that only 4 and 2% of the papers were assigned to the fields “Social Sciences” and “Arts & Humanities” is a significant finding. Considering highlighting this in the Abstract.

Thanks for the suggestion. The information is now considered in the abstract.

4. p.17, line 451: use of the word “local” languages seems inappropriate here. I imagine a lot has been published in Spanish, which is a global language. Would be good to clarify this.

The reviewer is right, we changed this in line XX (currently 494).

5. p.17, line 470: For synthesis reports, consider also citing the HKH Assessment report (Wester et al., 2019) and the Cross-Chapter Paper Mountains in IPCC AR6 WGII (Adler et al., 2022).

These references have now been added.

Note: references need to be carefully checked, e.g., the first reference by Adhikari, Shrestha and Shakya is incomplete.

Comments from Editor:

Among the other comments, please consider the points raised by reviewer 1 about the inclusion of an introductory paragraph in the methods section, and adding a map depicting mountain areas globally.

In addition to the reviewer reports I have a few points from an editor's perspective.

- The analysis of the vast amount of mountain literature over a long time period is impressive and allows you to produce multiple quantitative analyses. I think the analytical part and interpretation of the results in the bigger mountain research context could still be strengthened in the manuscript. I like how you address several aspects in this context in the Discussion and Conclusion section. However, I think there is more room for analytical text that goes beyond reporting the metrics and numbers of the quantitative analysis.

    1. For instance, what does this impressive literature analysis actually tell un in terms of status and development of mountain research?
    2. How has mountain research developed over this long time period, based on your results and additional consideration of literature?
    3. Is current mountain research fit to address the major challenges of today and the future?

    I'm aware that there is some limitation of going into much detail in this regard but I think some additional analysis would really strengthen the paper, also in view of the long-term experience and expertise of the author team. In this regard, please also consider and integrate the recently published mountain chapter in the IPCC AR6.

    We thank the editor for the impulse to more explicitly address these points and we have added respective statements and citations in the intro (63-64; 81-82), discussion (422-454) and conclusions (entire section).

- Technically, I think that figure visibility and readability should be improved, e.g. for Figs. 2 and 7 (in the latter one the blue color range is not visible), and possibly also for Figs. 3, 5, 6.

    The image quality was reduced during the automatic pdf generation in the PLOS submission system. Based on the original figures with high resolution or in vector format, we would be happy to optimize them in cooperation with the PLOS technical staff.

    One of the authors, whose mother tongue is English, has made a final language/style edit.

Journal Requirements:

When submitting your revision, we need you to address these additional requirements.

1. Please ensure that your manuscript meets PLOS ONE's style requirements, including those for file naming. The PLOS ONE style templates can be found at https://journals.plos.org/plosone/s/file?id=wjVg/PLOSOne_formatting_sample_main_body.pdf and https://journals.plos.org/plosone/s/file?id=ba62/PLOSOne_formatting_sample_title_authors_affiliations.pdf.

We hopefully managed to get the styles (mostly) correct.

2. In your Data Availability statement, you have not specified where the minimal data set underlying the results described in your manuscript can be found. PLOS defines a study's minimal data set as the underlying data used to reach the conclusions drawn in the manuscript and any additional data required to replicate the reported study findings in their entirety. All PLOS journals require that the minimal data set be made fully available. For more information about our data policy, please see http://journals.plos.org/plosone/s/data-availability.

Upon re-submitting your revised manuscript, please upload your study's minimal underlying data set as either Supporting Information files or to a stable, public repository and include the relevant URLs, DOIs, or accession numbers within your revised cover letter. For a list of acceptable repositories, please see http://journals.plos.org/plosone/s/data-availability#loc-recommended-repositories. Any potentially identifying patient information must be fully anonymized.

Important: If there are ethical or legal restrictions to sharing your data publicly, please explain these restrictions in detail. Please see our guidelines for more information on what we consider unacceptable restrictions to publicly sharing data: http://journals.plos.org/plosone/s/data-availability#loc-unacceptable-data-access-restrictions. Note that it is not acceptable for the authors to be the sole named individuals responsible for ensuring data access.

If needed, we would be happy to discuss with the editors if data beyond the one available in the supplement are desired. Importantly, all raw data can be reproduced by applying the search strings (S1 and S2 Tables) in the Web of Science Advanced search.

3. We note that Figure 7 and Striking Image in your submission contain [map/satellite] images which may be copyrighted. All PLOS content is published under the Creative Commons Attribution License (CC BY 4.0), which means that the manuscript, images, and Supporting Information files will be freely available online, and any third party is permitted to access, download, copy, distribute, and use these materials in any way, even commercially, with proper attribution. For these reasons, we cannot publish previously copyrighted maps or satellite images created using proprietary data, such as Google software (Google Maps, Street View, and Earth). For more information, see our copyright guidelines: http://journals.plos.org/plosone/s/licenses-and-copyright.

We require you to either (1) present written permission from the copyright holder to publish these figures specifically under the CC BY 4.0 license, or (2) remove the figures from your submission:

a) You may seek permission from the original copyright holder of Figure 7 and Striking Image to publish the content specifically under the CC BY 4.0 license.

We recommend that you contact the original copyright holder with the Content Permission Form (http://journals.plos.org/plosone/s/file?id=7c09/content-permission-form.pdf) and the following

text:
“I request permission for the open-access journal PLOS ONE to publish XXX under the Creative Commons Attribution License (CCAL) CC BY 4.0 (http://creativecommons.org/licenses/by/4.0/). Please be aware that this license allows unrestricted use and distribution, even commercially, by third parties. Please reply and provide explicit written permission to publish XXX under a CC BY license and complete the attached form.”

Please upload the completed Content Permission Form or other proof of granted permissions as an "Other" file with your submission.

In the figure caption of the copyrighted figure, please include the following text: “Reprinted from [ref] under a CC BY license, with permission from [name of publisher], original copyright [original copyright year].”

b) If you are unable to obtain permission from the original copyright holder to publish these figures under the CC BY 4.0 license or if the copyright holder’s requirements are incompatible with the CC BY 4.0 license, please either i) remove the figure or ii) supply a replacement figure that complies with the CC BY 4.0 license. Please check copyright information on all replacement figures and update the figure caption with source information. If applicable, please specify in the figure caption text when a figure is similar but not identical to the original image and is therefore for illustrative purposes only. The following resources for replacing copyrighted map figures may be helpful:

USGS National Map Viewer (public domain): http://viewer.nationalmap.gov/viewer/
The Gateway to Astronaut Photography of Earth (public domain): http://eol.jsc.nasa.gov/sseop/clickmap/
Maps at the CIA (public domain): https://www.cia.gov/library/publications/the-world-factbook/index.html and https://www.cia.gov/library/publications/cia-maps-publications/index.html
NASA Earth Observatory (public domain): http://earthobservatory.nasa.gov/
Landsat: http://landsat.visibleearth.nasa.gov/
USGS EROS (Earth Resources Observatory and Science (EROS) Center) (public domain): http://eros.usgs.gov/#
Natural Earth (public domain): http://www.naturalearthdata.com/

The data source of the background map is Natural Earth which doesn’t require any crediting. Nevertheless, we have added the source to the figure caption.

4. Please include captions for your Supporting Information files at the end of your manuscript, and update any in-text citations to match accordingly. Please see our Supporting Information guidelines for more information: http://journals.plos.org/plosone/s/supporting-information.

Captions are now provided at the end of the manuscript.

5. Please review your reference list to ensure that it is complete and correct. If you have cited papers that have been retracted, please include the rationale for doing so in the manuscript text, or remove these references and replace them with relevant current references. Any changes to the reference list should be mentioned in the rebuttal letter that accompanies your revised manuscript. If you need to cite a retracted article, indicate the article’s retracted status in the References list and also include a citation and full reference for the retraction notice.

Done and information updated.
