# Supplementary material for: Rising slopes—Bibliometrics of mountain research 1900–2019
Source: PLoS One. 2022 Aug 25;17(8):e0273421. doi: 10.1371/journal.pone.0273421 (PMC9409586; doi:10.1371/journal.pone.0273421)
Supplement: Attachment — Submitted filename: Peer Review of PONE-D-22-09392.docx [file pone.0273421.s010.docx]

**Peer Review of “Rising slopes – bibliometrics of mountain research 1900-2019”**

Excellent manuscript, highly relevant and well-developed. See below for suggested minor revisions:

p.1, line 24: replace “would be desirable” with “is recommended”.

p.2, lines 63-64: for comprehensive synthesis reports please consider also citing the HKH Assessment report (Wester et al., 2019) and the Cross-Chapter Paper Mountains in IPCC AR6 WGII (Adler et al., 2022).

p.9, lines 274- 276: that only 4 and 2% of the papers were assigned to the fields “Social Sciences” and “Arts & Humanities” is a significant finding. Considering highlighting this in the Abstract.

p.17, line 451: use of the word “local” languages seems inappropriate here. I imagine a lot has been published in Spanish, which is a global language. Would be good to clarify this.

p.17, line 470: For synthesis reports, consider also citing the HKH Assessment report (Wester et al., 2019) and the Cross-Chapter Paper Mountains in IPCC AR6 WGII (Adler et al., 2022).

Note: references need to be carefully checked, e.g., the first reference by Adhikari, Shrestha and Shakya is incomplete.
